# The prognostic value of semicircular canal function testing in idiopathic sudden sensorineural hearing loss: a systematic review and meta-analysis

**DOI:** 10.3389/fneur.2026.1796414

**Published:** 2026-04-10

**Authors:** Wu Zongyi, Zou Shizhen, Zhao Danheng, Yang Shuzhi, Diao Mingfang

**Affiliations:** Department of Otolaryngology-Head and Neck Surgery, Sixth Medical Center of PLA General Hospital, Beijing, China

**Keywords:** caloric test, hearing recovery, idiopathic sudden sensorineural hearing loss, prognosis, video head impulse test

## Abstract

**Purpose:**

This study aims to examine the prognostic value of the video head impulse test (vHIT) and caloric test in predicting hearing recovery with Idiopathic Sudden Sensorineural Hearing Loss (ISSHL).

**Methods:**

A comprehensive literature search was conducted across PubMed, Web of Science, Embase, China National Knowledge Infrastructure (CNKI), and Wanfang Data Knowledge Service Platform, covering studies published up to September 15, 2025. This meta-analysis included studies examining the association between vestibular function test parameters and hearing recovery outcomes. Data were extracted from eligible Chinese and English publications for a systematic review and meta-analysis of observational studies. This study protocol was registered with PROSPERO.

**Results:**

This meta-analysis included 781 patients from 7 studies. Normal function of the horizontal semicircular canal in vHIT (OR = 3.14, 95% CI: 1.71–5.77, *P* < 0.001, ***I^2^*** = 0.00%), normal function of the posterior semicircular canal in vHIT (OR = 6.929, 95% CI: 3.242–14.808, *P* < 0.001, ***I^2^*** = 0.00%), and normal caloric test results (OR = 3.184, 95% CI: 1.818–5.575, *P* < 0.001, ***I^2^*** = 0.00%) indicated a favorable prognosis for ISSHL patients. In contrast, normal function of the anterior semicircular canal in vHIT was not associated with prognosis in ISSHL patients (*P* = 0.186).

**Conclusion:**

vHIT, caloric tests, and Vestibular Evoked Myogenic Potentials (VEMP) can comprehensively evaluate vestibular function in patients with ISSHL. Normal vestibular function is a key factor for favorable hearing prognosis in these patients. Vestibular function testing should be performed as a routine examination in patients with ISSHL, as it provides a more comprehensive understanding of their hearing prognosis.

**Systematic review registration:**

https://www.crd.york.ac.uk/PROSPERO/view/CRD420251156585, Identifier: CRD420251156585.

## Background

Idiopathic sudden sensorineural hearing loss is defined as an unexplained sensorineural hearing loss of at least 20 dB HL across at least two adjacent frequencies, occurring within 72 h (1). The annual incidence ranges from 2 to 20 cases per 100,000 people (2). The pathogenesis of ISSHL remains unclear, with viral infection and vascular ischemia being the two most widely accepted hypotheses. The inner ear, composed of the cochlea and vestibular organs, receives its blood supply primarily from the labyrinthine artery. Damage to different segments of the artery can result in cochlear and/or vestibular symptoms (3). This shared blood supply suggests that vestibular impairment may reflect the severity of cochlear damage, thereby making vestibular function a potential prognostic indicator for hearing recovery in ISSHL. Due to their anatomical proximity and common blood supply, damage to one structure readily affects adjacent structures. Therefore, in ISSHL, vestibular dysfunction may coexist with cochlear injury. Even in the absence of vertigo symptoms, individuals with ISSHL may exhibit subclinical vestibular dysfunction. Such damage most commonly affects the semicircular canals but may also involve the utricle and saccule (4). Vestibular dysfunction associated with ISSHL was first documented in 1949 and is estimated to affect 30 to 40% of patients. Vertigo, a common manifestation of vestibular dysfunction, is typically described as a sensation of spinning (5). Acute vertigo episodes or balance problems occur in 28 to 57% of ISSHL patients (6).

Key prognostic factors for ISSHL include: age, delay in initiating treatment, presence of accompanying symptoms such as vertigo or tinnitus, underlying medical conditions, type of audiogram curve, and degree of hearing loss at onset (7). Evidence suggests that vestibular dysfunction is associated with poorer hearing outcomes, although clinical practice guidelines in various countries do not emphasize specialized testing for vestibular symptoms (8). A recent meta-analysis by González-García et al. (9), evaluated the impact of acute vestibular syndrome on hearing recovery in patients with ISSHL and examined the relationship between vestibular examination findings and hearing prognosis. The review found that patients with acute vestibular syndrome experienced poorer hearing recovery and higher rates of otolith organ involvement compared to those without acute vestibular syndrome. However, the study did not find a statistically significant correlation between caloric test results and hearing recovery. Furthermore, due to insufficient data, a meta-analysis of the vHIT could not be conducted, leaving a critical evidence gap regarding the high-frequency vestibular function assessed by this examination. To address these limitations and provide stronger evidence, the meta-analysis conducted a comprehensive search of Chinese and English databases to identify relevant literature. The meta-analysis objective is to present evidence-based findings elucidating the prognostic value of vHIT and caloric tests in predicting hearing recovery among patients with ISSHL.

## Methods

### Study design

This systematic review adheres to the Preferred Reporting Items for Systematic Reviews and Meta-Analyses (PRISMA) guidelines (10).

### Search strategy

**Figure 1:**
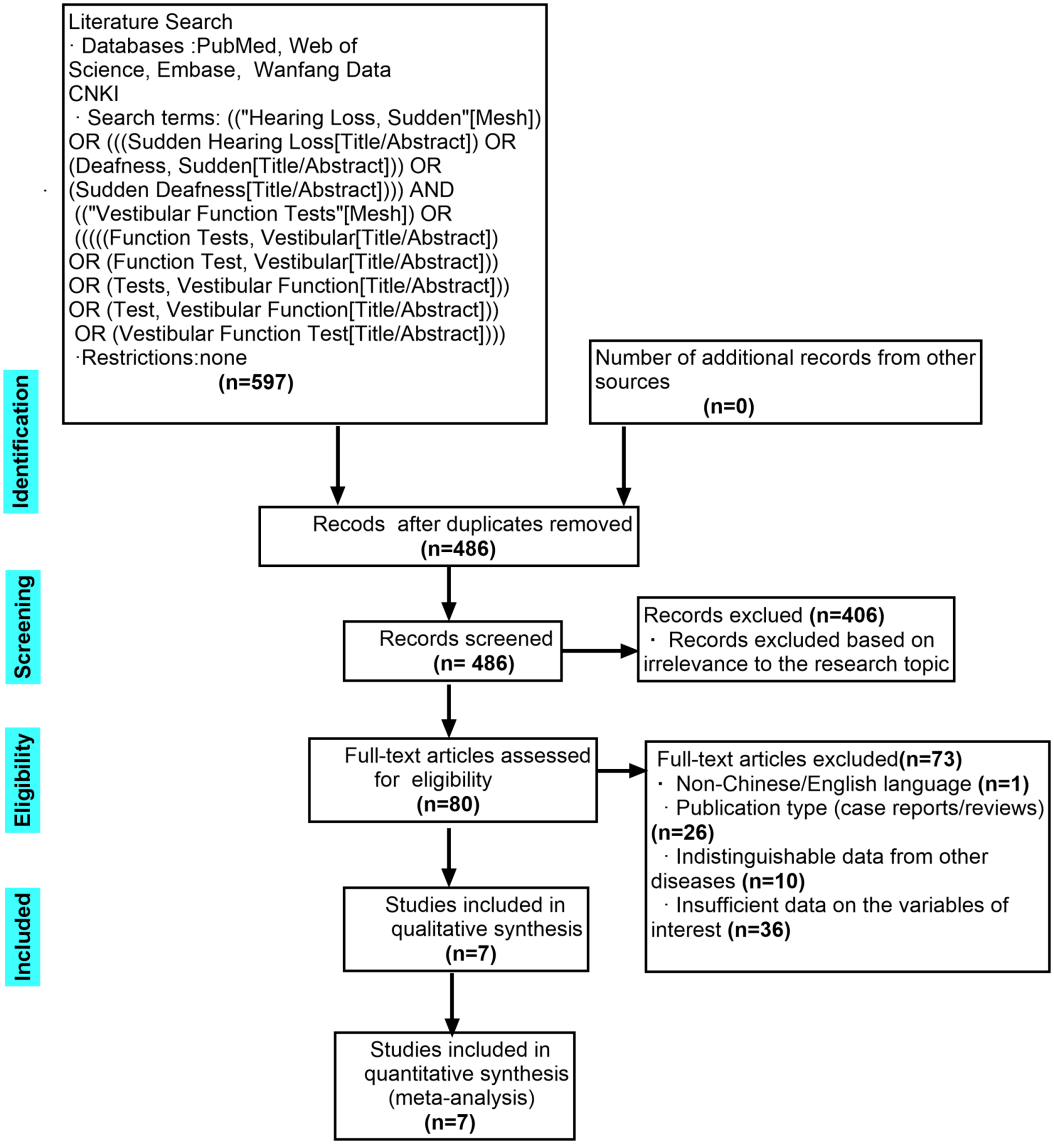
PRISMA flow diagram. PRISMA, preferred reporting items for systematic reviews and meta-analysis.

This meta-analysis performed an unrestricted search of PubMed, Web of Science, Embase, CNKI, and the Wanfang Database. Using a combination of database subject headings and free-text phrases, separate searches were conducted for the terms “vestibular test” and “sudden sensorineural hearing loss.” The two sets of search results were then combined using the Boolean operator “AND” (Figure 1). The search cutoff date was September 15, 2025. We screened all retrieved records by title and abstract to identify studies relevant to the research question. Full texts of potentially eligible studies were independently assessed by two reviewers (Wu Zongyi and Zou Shizhen) to determine preliminary inclusion. Disagreements were resolved through discussion. Additionally, the meta-analysis manually reviewed the reference lists of all retrieved articles.

### Inclusion and exclusion criteria

Inclusion criteria were determined based on the PICOT framework (i.e., population, intervention, control, outcome, and type of study).

Inclusion criteria:

- Study participants: Patients diagnosed with ISSHL.
- Intervention: Patients who underwent concurrent audiological and vestibular function examinations.
- Comparison: Hearing prognosis comparison between patients with normal versus abnormal vestibular function test results.
- Outcome measures: Outcomes include degree of hearing recovery determined by audiometric testing, and results from videonystagmography combined with caloric testing and videopulse testing.
- Study type: Eligible study designs are limited to retrospective studies, prospective studies, or clinical trials published in peer-reviewed, indexed journals.

Exclusion criteria:

- Publications not in Chinese or English.
- Case reports, review articles, and articles without full-text access.
- Studies where relevant data are not distinguished from data on other diseases.
- Studies lacking required variable data.

### Data extraction and quality assessment

The meta-analysis extracted the following data from studies meeting inclusion/exclusion criteria:

- Study characteristics: Publication year, study location, study design (prospective or retrospective), sample size.
- Outcomes: Complete recovery, partial recovery, no recovery.
- vHIT parameters: Horizontal semicircular canal parameters, anterior semicircular canal parameters, posterior semicircular canal parameters.

### Caloric test results: normal or abnormal

- Methodological quality and risk of bias were assessed using the MINORS (Methodological Index for Non-Randomized Studies) criteria (11). For non-comparative studies, quality was graded as: Very low (0-4 points), Low (5-8 points), Moderate (9-12 points), or High (13-16 points). For comparative studies, the grading criteria are: very low (0-6 points), low (7-12 points), moderate (13-18 points), or high (19-24 points), following the framework proposed by Khan et al. (12).

### Statistical analysis

**Figure 2:**
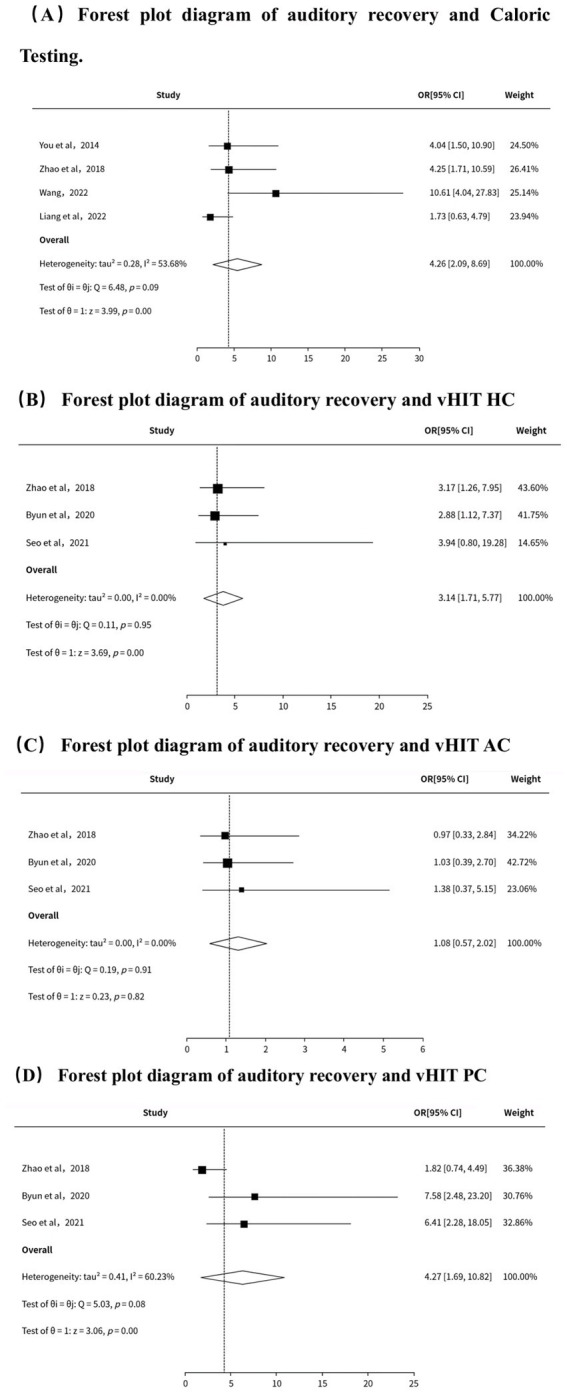
Forest plot diagram of auditory recovery and vestibular function.

The meta-analysis was conducted using the web-based SPSSAU software, with results presented in forest plot format. Heterogeneity was assessed using the ***I^2^*** statistic (13); when ***I^2^*** = 0, a random-effects model is used; when ***I^2^*** > 0, a fixed-effects model is employed. For analyses showing significant heterogeneity (***I^2^*** > 50%), the random-effects model was applied to determine whether abnormal vHIT parameters or abnormal caloric test results correlate with reduced odds of hearing recovery. Due to the limited number of included studies, subgroup analyses to explore the sources of heterogeneity could not be conducted (Figure 2).

## Results

### Literature search

The initial search identified a total of 597 records. Figure 1 details the study screening process. After removing duplicate publications and excluding records that did not meet the inclusion criteria or for which full-text access was unavailable, seven studies were ultimately included in this meta-analysis (14–20).

### Characteristics of the included studies

Table 1 summarizes the characteristics of the seven included studies. The overall low MINORS scores indicated generally poor study quality, primarily attributable to the retrospective design of all included studies and the lack of long-term follow-up. Among these, three studies from China defined hearing recovery based on the 2015 Chinese Guidelines for Diagnosis and Treatment of Otolaryngology-Head and Neck Surgery (PTA < 20 dB HL) (15, 19, 20). One Chinese study applied criteria from a 1988 publication (PTA < 20 dB HL) (14, 21). Another Chinese study did not specify the hearing recovery criteria used (17). The two Korean studies used the Siegel Criteria (PTA < 25 dB HL) (16), and the AAO-HNS criteria (PTA < 20 dB HL) (18), respectively.

**Table 1:** Characteristics of included studies.

Author	Year	Location	Design	Patients	Ears	MINORS score	Hearing recovery criteria	Definition of abnormal vHIT
You et al. (14)	2014	China	Retrospective	75	75	8	PTA < 20 dB HL	None
Zhao et al. (15)	2018	China	Retrospective	154	NR	8	PTA < 20 dB HL	The VOR gain values of the three semicircular canals are <0.79, or saccadic waves are present.
Byun et al. (16)	2020	Korea	Retrospective	148	148	7	PTA < 25 dB HL	A calculated VOR gain of <0.7 was considered abnormal.
Guan et al. (17)	2020	China	Retrospective	59	59	6	None	The gain of horizontal semicircular canal is <0.8 and that of vertical semicircular canal is <0.7
Seo et al. (18)	2021	Korea	retrospective	147	147	8	PTA < 20 dB HL	The gain of horizontal semicircular canal is <0.8 and that of vertical semicircular canal is <0.7
Wang (20)	2022	China	retrospective	134	134	8	PTA < 20 dB HL	The gain of horizontal semicircular canal is <0.8 and that of vertical semicircular canal is <0.7
Liang et al. (19)	2022	China	retrospective	64	64	6	PTA < 20 dB HL	None

PTA, pure-tone audiometry; None, not reported; VOR,Vestibulo-Ocular Reflex.

### The prognostic value of caloric testing and vHIT for ISSHL

This meta-analysis included 781 patients from 7 studies. Results showed that vHIT demonstrated normal function of the horizontal semicircular canal (OR = 3.14, 95% CI: 1.71–5.77, *P* < 0.001, ***I^2^*** = 0.00%), normal function of the posterior semicircular canal (OR = 6.929, 95% CI: 3.242–14.808, *P* = 0.002, ***I^2^*** = 0.00%), and normal Caloric test results (OR = 4.26, 95% CI: 3.06–10.42, *P* < 0.001, ***I^2^*** = 53.68%) were all associated with a favorable prognosis in patients with ISSHL. In contrast, normal anterior semicircular canal function on vHIT was not associated with prognosis in ISSHL patients (*P* = 0.186). Among ISSHL patients, those with normal horizontal semicircular canal function on vHIT demonstrated superior hearing recovery compared to those with abnormal function (mean difference = 0.640, 95% CI: 0.112–1.167, *P* = 0.017, ***I^2^*** = 0.00%). Similarly, those with normal Caloric test results showed superior hearing recovery compared to those with abnormal results (mean difference = 0.84, 95% CI: 0.47–1.21, *P* = 0.001, ***I^2^*** = 0.00%) (Table 2).

**Table 2:** Meta-analysis of vestibular function and hearing improvement.

Study	Normal (*n*)		Abnormal (*n*)		Odds ratio or mean difference, 95%CI	*I^2^*	*p*
	vHIT HC		vHIT HC				
	Events	Total	Events	Total			
Categorical variable							
Zhao et al. (15)	38	56	12	30	3.17 [1.26, 7.95]		
Byun et al. (16)	66	125	7	25	2.88 [1.12, 7.37]		
Seo et al. (18)	62	125	2	10	3.94 [0.80, 19.28]		
Total		306		65	3.14 [1.71, 5.77]	0.00%	**<0.001**

Study	Normal (*n*)		Abnormal (*n*)		Odds ratio or mean difference, 95%CI	*I^2^*	*p*
	vHIT AC		vHIT AC				
	Events	Total	Events	Total			
Categorical variable							
Zhao et al. (15)	40	69	10	17	0.97 [0.33, 2.84]		
Byun et al. (16)	62	129	9	19	1.03 [0.39, 2.70]		
Seo et al. (18)	60	125	4	10	1.38 [0.37, 5.15]		
Total		323		46	1.08 [0.57, 2.02]	0.00%	0.816

Study	Normal (*n*)		Abnormal (*n*)		Odds ratio or mean difference, 95%CI	*I^2^*	*p*
	vHIT PC		vHIT PC				
	Events	Total	Events	Total			
Categorical variable							
Zhao et al. (15)	34	23	13	29	1.82 [0.74, 4.49]		
Byun et al. (16)	67	120	4	28	7.58 [2.48, 23.20]		
Seo et al. (18)	59	105	5	30	6.41 [2.28, 18.45]		
Total		248		87	4.27 [1.69, 10.82]	60.23%	**0.002**

Study	Normal (*n*)		Abnormal (*n*)		Odds ratio or mean difference, 95%CI	*I^2^*	*p*
	Caloric tests		Caloric tests				
	Events	Total	Events	Total			
Categorical variable							
You et al. (14)	34	47	11	28	4.32 [1.59, 11.73]		
Zhao et al. (15)	34	46	16	40	4.24 [1.62, 11.88]		
Wang (20)	60	93	6	41	3.16 [1.82, 5.58]		
Liang et al. (19)	13	25	15	39	5.65 [3.06, 10.42]		
Total		211		148	4.26 [3.06, 10.42]	53.68%	**<0.001**

Study	Normal (*n*)			Abnormal (*n*)			Odds ratio or mean difference, 95%CI	*I^2^*	*p*
	vHIT HC			vHIT HC					
	Mean	SD	Total	Mean	SD	Total			
Continuous variables									
Guan et al. (17)	24.65 33	19.12	22	14.15 26	11.41	26	0.640 [0.112, 1.167]	0.00%	**0.017**

Study	Normal (*n*)			Abnormal (*n*)			Odds ratio or mean difference, 95%CI	*I^2^*	*p*
	vHIT AC			vHIT AC					
	Mean	SD	Total	Mean	SD	Total			
Continuous variables									
Guan et al. (17)	21.65	17 53	55	8.12	6.65	4	0.779 [−0.246, 1.805]	0.00%	0.136

Study	Normal (*n*)			Abnormal (*n*)			Odds ratio or mean difference, 95%CI	*I^2^*	*p*
	vHIT PC			vHIT PC					
	Mean	SD	Total	Mean	SD	Total			
Continuous variables									
Guan et al. (17)	23.00	19.09	33	15.98	11.95	26	0.429 [−0.096, 0.944]	0.00%	0.110

Study	Normal (*n*)			Abnormal (*n*)			Odds ratio or mean difference, 95%CI	*I^2^*	*p*
	Caloric tests			Caloric tests					
	Mean	SD	Total	Mean	SD	Total			
Continuous variables									
Guan et al. (17)	27.61	19.47	27	14.94	12.95	32	0.77 [0.24, 1.30]		
Liang et al. (19)	32.55	19.56	25	15.29 19	18.19	19	0.9 [0.38, 1.44]		
Total			52			51	0.84 [0.47, 1.21]	0.00%	**<0.001**

Bold indicates statistically significant.

### Sensitivity analysis

As shown in Table 3, sensitivity analysis of hearing recovery based on caloric tests revealed that excluding the study by Wang et al. (20) significantly reduced statistical heterogeneity (OR = 3.184, 95% CI: 1.818–5.575, *P* < 0.001, ***I^2^*** = 0.00%), with no apparent change in effect size, indicating robust results. Similarly, Table 3 shows that for posterior semicircular canal vHIT, excluding the study by Zhao et al. also resulted in significantly reduced heterogeneity (OR = 0.143, 95% CI: 3.424–14.808, *P* < 0.001, ***I^2^*** = 0.00%), with no substantial change in effect size, further confirming the robustness of the results (Table 4).

**Table 3:** Sensitivity analysis of hearing recovery based on caloric test.

Study	OR	95%CI	*p*	*I^2^* (%)
You et al. (14)	4.317	1.588, 11.732	**0.004**	69.03
Zhao et al. (15)	4.245	1.516, 11.885	**0.006**	69.14
Wang (20)	3.184	1.818, 5.575	**<0.001**	0.00%
Liang et al. (19)	5.647	3.061, 10.419	**<0.001**	18.91%

**Table 4:** Sensitivity analysis of hearing recovery based on vHIT for the PC.

Study	OR	95%CI	*p*	*I^2^* (%)
Zhao et al. (15)	6.929	3.242, 14.808	**<0.001**	0.00%
Byun et al. (16)	3.328	0.964, 11.490	0.057	69.35
Seo et al. (18)	3.573	0.873, 14.617	0.076	60.23

## Discussion

This meta-analysis provides strong evidence that normal results in Caloric tests and vHIT (particularly for the horizontal and posterior semicircular canals) are significant predictors of favorable hearing recovery in patients with ISSHL. This finding supports the clinical utility of vestibular function assessment in these patients.

This study conducted a comprehensive search of Chinese and English databases to address research gaps not covered in previous reviews (9). It is the first to demonstrate, through meta-analysis, the prognostic value of the vHIT in assessing horizontal and posterior semicircular canal function. Meta-analysis results indicate: Among patients with ISSHL, those with normal caloric test results had a 3.184-fold higher probability of hearing recovery compared to those with abnormal results. Patients with normal horizontal semicircular canal function on vHIT had a 3.14-fold higher probability of hearing recovery than those with abnormal function. Notably, patients with normal posterior semicircular canal function on vHIT demonstrated an even higher probability of hearing recovery, reaching 6.929 times that of those with abnormal function. However, no significant statistical association was found between abnormal anterior semicircular canal function on vHIT and hearing recovery.

The function of the semicircular canals exhibits frequency specificity. Both type I and type II hair cells coexist within the crista ampullaris. The central region of the crista is predominantly populated by type I hair cells, which primarily transmit high-frequency impulses and exhibit a high spontaneous discharge frequency. Type II hair cells are mainly distributed in the peripheral region of the crista ampullaris, primarily responding to low-frequency stimuli with a lower spontaneous discharge frequency. By assessing the high-frequency function of the semicircular canals, vHIT can identify whether the superior or inferior vestibular nerves are affected by high-frequency damage (22, 23). The vHIT is a relatively new method for assessing vestibulo-ocular reflex function within the natural frequency range of the three semicircular canals (24). vHIT provides high-frequency (4–5 Hz) information about vestibular system function, which more closely approximates the frequency range of natural head movements compared to traditional vestibular function testing. As noted in one study (17), this makes vHIT a crucial complement to the caloric test, which operates at a low frequency (0.01–0.025 Hz). Research has found that patients with abnormal cervical vestibular-evoked myogenic potentials to air-conducted sound are 3.22 times more likely to have poorer hearing recovery (9). There is a dissociation between the results of the caloric test and vHIT (25). Combined use of VEMP, caloric tests, and vHIT enables comprehensive assessment of vestibular function in patients with ISSHL and can be applied to predict their hearing prognosis.

The labyrinthine artery is primarily divided into the anterior vestibular artery and the cochlear artery. The cochlear artery further branches into the vestibulocochlear artery and the spiral cochlear artery. The posterior vestibular branch of the vestibulocochlear artery supplies the utricle, the lower part of the saccule, and the posterior semicircular canal. The anterior vestibular artery primarily supplies the utricle, the upper part of the saccule, the anterior semicircular canal, and the lateral semicircular canal (26). The blood supply and innervation patterns of the vestibule and horizontal semicircular canals differ: the horizontal semicircular canal is supplied by the anterior vestibular artery and innervated by the superior vestibular nerve; the posterior semicircular canal, however, is supplied by the vestibulocochlear artery and innervated by the inferior vestibular nerve (16). However, studies have revealed that the patterns of vestibular end-organ dysfunction exhibit high variability and do not always correspond to specific neural or vascular innervation patterns. For instance, the horizontal semicircular canal may retain normal function while the anterior semicircular canal shows impairment; alternatively, all semicircular canals may remain intact, yet all otolith organs may be compromised (18). Consistent with this variability, the meta-analysis found that abnormal horizontal semicircular canal function in vHIT has predictive value, whereas abnormal anterior semicircular canal function does not. The anterior semicircular canal is supplied by the anterior vestibular artery, which has relatively fine branches but a more abundant collateral circulation. This anatomy may explain why damage to this structure results in less severe overall impact on the inner ear. These anatomical differences in vascular supply and collateral circulation could potentially account for why abnormal anterior semicircular canal function detected by vHIT is not a significant prognostic marker. Furthermore, the sample size of patients with anterior semicircular canal abnormalities in the included studies was small, and limited statistical power may have obscured potential correlations. The absence of clear anatomical correlations between vestibular damage patterns suggests that vestibular dysfunction and hearing loss may result from ischemia or other conditions such as viral labyrinthitis, autoimmune labyrinthopathy, tympanic membrane rupture, or specific vulnerability of particular hair cell/otolith organ populations.

A limitation of the current study is that its conclusion—that peripheral semicircular canal dysfunction predicts prognosis—was drawn without considering central compensatory mechanisms. Future research should simultaneously evaluate central compensatory capacity to more accurately predict outcomes. The presence of spontaneous nystagmus has been found to influences outcomes in ISSHL (8). Furthermore, current studies lack data on vestibular function recovery, making it impossible to comprehensively evaluate the relationship between vestibular function and hearing recovery. Previous research indicates that for patients with ISSHL accompanied by vertigo, there is no clear association between vestibular function recovery and either vertigo symptom improvement or hearing recovery. However, these studies primarily focused on patients with ISSHL accompanied by vertigo and assessed vestibular recovery within 60 days, which may be insufficient to represent a final and stable state (6). Nearly half of the patients experienced hearing recovery within the first 2 weeks, and after 3 months, nearly all patients achieved stable, permanent hearing levels (27). Accordingly, another study found that recovery rates at 3 months post-onset were identical to those at 1 year, with hearing recovery reaching a distinct plateau at the 3-month mark (3).

Several limitations of the study warrant consideration. All included studies employed retrospective designs, which may introduce selection bias and recall bias.

Significant heterogeneity exists across studies, stemming from differences in vestibular detection techniques, definitions of hearing recovery, and timing of auditory assessments. Therefore, the results of this meta-analysis should be interpreted with caution, particularly given the relatively small sample sizes in some of the included analyses. Since the data in existing published literature are based solely on qualitative classifications (normal/abnormal) without analyzing the degree of vHIT gain reduction or the percentage of Canal Paresis values in caloric tests, it is not possible to more accurately predict the prognosis for patients with ISSHL. Since all studies included in this systematic review originated solely from China and South Korea, with no Western or other regional studies identified, its conclusions may not be generalizable to broader populations. This underscores the necessity for future validation studies involving more diverse populations. Caloric tests and vHIT assess vestibular function at different frequencies. Some current studies fail to perform comprehensive vestibular function examinations simultaneously within the same patient cohort, typically conducting only partial vestibular assessments per patient. Consequently, when results from these two tests are inconsistent, such studies cannot reflect the prognosis for patients with ISSHL. Future studies should adopt a comprehensive evaluation approach in patients with ISSHL, integrating caloric tests, vHIT, and VEMP to analyze the prognostic value of these tests. Furthermore, the varying definitions of vHIT abnormalities across included studies contribute to the high heterogeneity observed in the meta-analysis. Finally, due to the lack of information regarding vestibular recovery itself, the meta-analysis cannot accurately understand the relationship between vestibular recovery and hearing recovery over time.

Future studies should incorporate long-term follow-up to establish prognostic models for ISSHL and calculate odds ratios for key parameters to investigate the impact of central compensation on prognosis. Studies conducting longitudinal follow-up for at least the first 3 months after symptom onset will enable comprehensive assessment of prognosis in patients with ISSHL. For patients with ISSHL without vertigo, vestibular function assessment should also be prioritized. Vestibular testing may reveal diverse patterns of abnormalities. While current studies often categorize findings simplistically as “normal” or “abnormal,” future research should delve into the prognostic significance of specific abnormal parameters themselves.

## Conclusion

vHIT, caloric tests, and VEMP can be used to comprehensively evaluate vestibular function in patients with ISSHL. Normal vestibular function is a key factor for favorable hearing prognosis in these patients. Vestibular function testing should be performed as a routine examination in patients with ISSHL, as it provides a more comprehensive understanding of their hearing prognosis.

## Data Availability

The original contributions presented in the study are included in the article/supplementary material, further inquiries can be directed to the corresponding author.

## References

[1] Editorial Board of Chinese Journal of Otorhinolaryngology Head and Neck Surgery; Chinese Medical Association Otorhinolaryngology Head and Neck Surgery Branch. Guidelines for the diagnosis and treatment of sudden sensorineural hearing loss. Chin J Otorhinolaryngol Head Neck Surg. (2015) 50:443–7. doi: 10.3760/cma.j.issn.1673-0860.2015.06.002

[2] Schreiber BE Agrup C Haskard DO Luxon LM. Sudden sensorineural hearing loss. Lancet. (2010) 375:1203–11. doi: 10.1016/s0140-6736(09)62071-7, 20362815

[3] Hepkarsi S Kaya I Kirazli T. Vestibular function assessment in idiopathic sudden sensorineural hearing loss: a prospective study. Eur Arch Otorrinolaringol. (2024) 281:2365–72. doi: 10.1007/s00405-023-08361-7, 38095708

[4] Liu RQ Zhang Y Liu B. Research status of vestibular function assessment in patients with sudden sensorineural hearing loss. Chin J Otol (2024) 22:14–18. Available online at: https://oversea.cnki.net/index/ (Accessed December 28, 2025).

[5] Battat N Ungar OJ Handzel O Eta RA Oron Y. Video head impulse test for the assessment of vestibular function in patients with idiopathic sudden sensorineural hearing loss without vertigo. J Laryngol Otol. (2023) 137:1374–7. doi: 10.1017/s0022215123000245, 36794537 PMC10694636

[6] Hao W Ye L Yu H Li H. Prognosis of vestibular dysfunction in idiopathic sudden sensorineural hearing loss with vertigo: a prospective cohort study. J Neurol. (2023) 270:5516–26. doi: 10.1007/s00415-023-11894-w, 37517037

[7] Perez Ferreira Neto A. da Costa Monsanto R. Dore Saint Jean L. Sonzzini Ribeiro Souza L. Oliveira Penido N. Clinical profile of patients with unilateral sudden sensorineural hearing loss: correlation with hearing prognosis (2021). Otolaryngol Head Neck Surg, 165: 563–570 doi: 10.1177/0194599820986571 33557702

[8] Qian Y Kang H Zhong S Tao C Zuo W Lei Y . The role of asymmetry values, gain, and pathological saccades of the video head impulse test (vHIT) in sudden sensorineural hearing loss. Otol Neurotol. (2024) 45:e509–16. doi: 10.1097/mao.0000000000004247, 38918071

[9] González-García M Prieto-Sánchez-de-Puerta L Domínguez-Durán E Sánchez-Gómez S. Auditory prognosis of patients with sudden sensorineural hearing loss in relation to the presence of acute vestibular syndrome: a systematic literature review and Meta-analysis. Ear Hear. (2025) 46:8–15. doi: 10.1097/aud.0000000000001576, 39252156

[10] Page MJ McKenzie JE Bossuyt PM Boutron I Hoffmann TC Mulrow CD . The PRISMA 2020 statement: an updated guideline for reporting systematic reviews. Syst Rev. (2021) 10:89. doi: 10.1186/s13643-021-01626-4, 33781348 PMC8008539

[11] Slim K Nini E Forestier D Kwiatkowski F Panis Y Chipponi J. Methodological index for non-randomized studies (minors): development and validation of a new instrument. ANZ J Surg. (2003) 73:712–6. doi: 10.1046/j.1445-2197.2003.02748.x, 12956787

[12] Khan W Khan M Alradwan H Williams R Simunovic N Ayeni OR. Utility of intra-articular hip injections for Femoroacetabular impingement: a systematic review. Orthop J Sports Med. (2015) 3:2325967115601030. doi: 10.1177/2325967115601030, 26535395 PMC4622294

[13] Higgins JP Thompson SG Deeks JJ Altman DG. Measuring inconsistency in meta-analyses. BMJ. (2003) 327:557–60. doi: 10.1136/bmj.327.7414.557, 12958120 PMC192859

[14] You TZ Wang SJ Young YH. Registering grades of sudden deafness to predict the hearing outcome via an inner-ear test battery. Int J Audiol. (2014) 53:153–8. doi: 10.3109/14992027.2013.851798, 24286348

[15] Zhao Z Luo B Guan R Liu X W W Sun J . Effect of vestibular function on the prognosis of sudden deafness. J Audiol Speech Pathol. (2018) 26:237–42. doi: 10.3969/j.issn.1006-7299.2018.03.003

[16] Byun H Chung JH Lee SH. Clinical implications of posterior semicircular canal function in idiopathic sudden sensorineural hearing loss. Sci Rep. (2020) 10:8313. doi: 10.1038/s41598-020-65294-5, 32433568 PMC7239936

[17] Guan R Zhao Z Guo X Sun J. The semicircular canal function tests contribute to identifying unilateral idiopathic sudden sensorineural hearing loss with vertigo. Am J Otolaryngol. (2020) 41:102461. doi: 10.1016/j.amjoto.2020.102461, 32201018

[18] Seo HW Chung JH Byun H Lee SH. Vestibular mapping assessment in idiopathic sudden sensorineural hearing loss. Ear Hear. (2021) 43:242–9. doi: 10.1097/AUD.0000000000001129, 34524151

[19] Liang M Wu H Chen JY Zhang Q Li SA Zheng GL . Vestibular evoked myogenic potential may predict the hearing recovery in patients with unilateral idiopathic sudden sensorineural hearing loss. Front Neurol. (2022) 13:1017608. doi: 10.3389/fneur.2022.1017608, 36408508 PMC9666675

[20] Wang YX. Correlation Analysis of Vestibular Function and Electrocochleography With the Prognosis of Sudden Sensorineural Hearing Loss [master’s thesis]. Yan'an City: Yan'an University (2022).

[21] Nomura Y. Diagnostic criteria for sudden deafness, mumps deafness and perilymphatic fistula. Acta Otolaryngol Suppl. (1988) 456:7–8. doi: 10.3109/00016488809125068, 3227833

[22] Cen J Zhang S Yuan T Liang Y Zeng X. A preliminary study on semicircular canal function assessment by video head impulse test and caloric test. Chin J Otol. (2018) 16:267–71. doi: 10.3969/j.issn.1672-2922.2018.03.002

[23] Hou LX Chen TS Xu KX Wang W Li SS Liu Q . Evaluation of the injured range of vestibular superior and inferior nerves in sudden deafness patients with vertigo using video head impulse test. Chin J Otorhinolaryngol Head Neck Surg. (2015). 50:718–722. 26696342

[24] Liu Y Leng Y Zhou R Liu J Wang H Xia K . Video head impulse test findings in patients with benign paroxysmal positional vertigo secondary to idiopathic sudden sensorineural hearing loss. Front Neurol. (2022) 13:877777. doi: 10.3389/fneur.2022.877777, 35720082 PMC9202345

[25] Hu JJ Wang CC Tong JB Gong FY Jin ZG Jia HB. Performance of video head impulse test in patients with bilateral vestibular weakness in the caloric test. Med J Air Force. (2025) 42:28–31. doi: 10.3969/j.issn.2097-1753.2025.01.007

[26] Gu R Jiang SC Wang ZM. Otology. 2nd ed. Shanghai: Shanghai Scientific and Technical Publishers (2002).

[27] Jiang Z Zhang J Wang Y Huang X Yao Q Feng Y . Contribution of audiogram classification in evaluating vestibular dysfunction in sudden sensorineural hearing loss with vertigo. Front Neurol. (2021) 12:667804. doi: 10.3389/fneur.2021.667804, 33995264 PMC8116712

